# Markedly increased ocular side effect causing severe vision deterioration after chemotherapy using new or investigational epidermal or fibroblast growth factor receptor inhibitors

**DOI:** 10.1186/s12886-019-1285-9

**Published:** 2020-01-09

**Authors:** Eunhae Shin, Dong Hui Lim, Jisang Han, Do-Hyun Nam, Keunchil Park, Myung-Ju Ahn, Won Ki Kang, Jeeyun Lee, Jin Seok Ahn, Se-Hoon Lee, Jong-Mu Sun, Hyun Ae Jung, Tae-Young Chung

**Affiliations:** 1 Department of Ophthalmology, Samsung Medical Center, Sungkyunkwan University School of Medicine, Seoul, Republic of Korea; 2 Department of Preventive Medicine, Graduate School, Catholic University of Korea, Seoul, Republic of Korea; 3 Department of Ophthalmology, Kangbuk Samsung Hospital, Sungkyunkwan University School of Medicine, Seoul, Republic of Korea; 4 Cancer Stem Cell Research Center, Department of Neurosurgery, Samsung Medical Center and Samsung Biomedical Research Institute, Sungkyunkwan University School of Medicine, Seoul, Republic of Korea; 5 Division of Hematology-Oncology, Department of Medicine, Samsung Medical Center, Sungkyunkwan University School of Medicine, Seoul, Republic of Korea

**Keywords:** EGFR inhibitor, FGFR inhibitor. Vortex keratopathy, Corneal epithelial change

## Abstract

**Background:**

We sought to describe corneal epithelial changes after using epidermal (EGFR) or fibroblast growth factor receptor (FGFR) inhibitors as chemotherapy and to clarify incidence and prognosis.

**Materials:**

Retrospective chart review.

**Results:**

Among 6871 patients and 17 EGFR or FGFR inhibitors, 1161 patients (16.9%) referred for ophthalmologic examination. In total, 1145 patients had disease-related or unrelated ocular complications. Among 16 patients with treatment-related ocular complications, three patients had treatment-related radiation retinopathy and one patient showed treatment-related corneal ulcer. Finally the authors identified that, in 12 patients, three EGFR inhibitors and two FGFR inhibitors caused corneal epithelial lesions. Vandetanib, Osimertinib, and ABT-414 caused vortex keratopathy in nine patients, while ASP-5878 and FPA-144 caused epithelial changes resembling corneal dysmaturation in three patients. The mean interval until symptoms appeared was 246 days with vandetanib, 196 days with osimertinib, 30 days with ABT-414, 55 days with ASP-5878, and 70 days with FPA-144. The mean of the lowest logarithm of minimal angle of resolution visual acuity results of the right and left eyes after chemotherapy were 0.338 and 0.413. The incidence rates of epithelial changes were 15.79% with vandetanib, 0.5% with osimertinib, 100% with ABT-414, 50.0% with ASP-5878, and 18.2% with FPA-144. After excluding deceased patients and those who were lost to follow-up or still undergoing treatment, we confirmed the reversibility of corneal lesions after the discontinuation of each agent. Seven patients showed full recovery of their vision and corneal epithelium, while three achieved a partial level of recovery. Although patients diagnosed with glioblastoma used prophylactic topical steroids before and during ABT-414 therapy, all developed vortex keratopathy.

**Conclusions:**

EGFR and FGFR inhibitors are chemotherapy agents that could make corneal epithelial changes. Contrary to the low probability of ocular complication with old EGFR drugs, recently introduced EGFR and FGFR agents showed a high incidence of ocular complication with severe vision distortion. Doctors should forewarn patients planning chemotherapy with these agents that decreased visual acuity could develop due to corneal epithelial changes and also reassure them that the condition could be improved after the end of treatment without the use of steroid eye drops.

**Trial registration:**

This study was approved by the institutional review board (IRB) of Samsung Medical Center (IRB no. 2019–04-027) and was conducted according to the principles expressed in the Declaration of Helsinki.

## Background

Epidermal growth factor (EGF) receptor (EGFR), a member of the ErbB family of receptor tyrosine kinases [1], is a transmembrane protein activated by EGF and EGF-like molecules that affects deoxyribonucleic acid synthesis, cell differentiation, cell migration, cell mitosis, and cell apoptosis [2]. Fibroblast growth factor (FGF) receptor (FGFR)s are a family of four transmembrane receptor tyrosine kinases activated by FGF that mediate tissue and metabolism homeostasis, endocrine function, and wound repair [3]. The overexpression of EGFR or FGFR results in an abnormal proliferation of cancer cells. Both receptors are overexpressed in cancers such as nonsmall-cell lung cancer, glioblastoma, head and neck squamous cell carcinoma, hepatocellular cell carcinoma, colorectal and pancreatic cancer [1, 3, 4].

The EGFR and FGFR systems play a key role in the cornea in cell proliferation, differentiation, tear film secretion and the recovery of corneal epithelial damage [2]. Chemotherapy agents interfering with EGFR or FGFR pathways have known ocular side effects such as acquired trichomegaly, persistent corneal epithelial defects, dysfunctional tear syndrome, blepharitis, meibomitis, iridocyclitis, and lid ectropion, etc. [1, 2, 4] However, recent articles have reported the appearance of epithelial changes after EGFR inhibitor chemotherapy, including vortex keratopathy, a whorl-like pattern of corneal haziness [5, 6]. Unfortunately, those reports contain few cases and lack long-term follow-up data, making recovery difficult to determine. Furthermore, many doctors are unaware of this kind of side effect.

There are various kinds of new or investigational chemotherapy drugs capable of inhibiting EGFR or FGFR, such as ABT-414 (depatuxizumab mafodotin, 1.25 mg/kg, intravenous infusion; AbbVie, Chicago, IL, USA), an investigational compound that targets a tumor-selective EGFR epitope [7]; ASP-5878 (2 mg, oral twice daily; Astellas Pharma Inc., Tokyo, Japan), a novel drug that inhibits all FGFRs [8]; and FPA-144 (bemarituzumab, 15 mg/kg, intravenous infusion; Five Prime Therapeutics, Inc., San Francisco, CA, USA), an enhanced monoclonal antibody against FGFR2b [9]. Recently, we have encountered quite a few cases of corneal epithelial changes different from known ocular side effects in a retrospective chart review of patients who were treated with these new compounds. Here, we report that EGFR and FGFR inhibitors can cause corneal epithelial changes, including vortex keratopathy, which mimic corneal dysmaturation.

## Methods

This was a retrospective study using medical chart review performed at Samsung Medical Center, Seoul, Republic of Korea. All patients who received any kind of chemotherapy using an EGFR or FGFR inhibitor between November 1994 and August 2017 were reviewed. Among them, we analyzed patients with a history of ophthalmologic examination. Cases were defined when the patient had regular ophthalmologic records describing features of corneal epithelial lesions consecutively. Patients with simple punctate epithelial erosions or dry eye syndrome without photographic data were not included as cases. We calculated the time lapse between the start of chemotherapy and diagnosis of corneal change (days), the time interval between the end of chemotherapy and corneal recovery (days), and the amount of agents administered to evaluate the dose–response relationship. This study was approved by the institutional review board (IRB) of Samsung Medical Center (IRB no. 2019–04-027) and was conducted according to the principles expressed in the Declaration of Helsinki.

## Results

### General characteristics

**Fig. 1:**
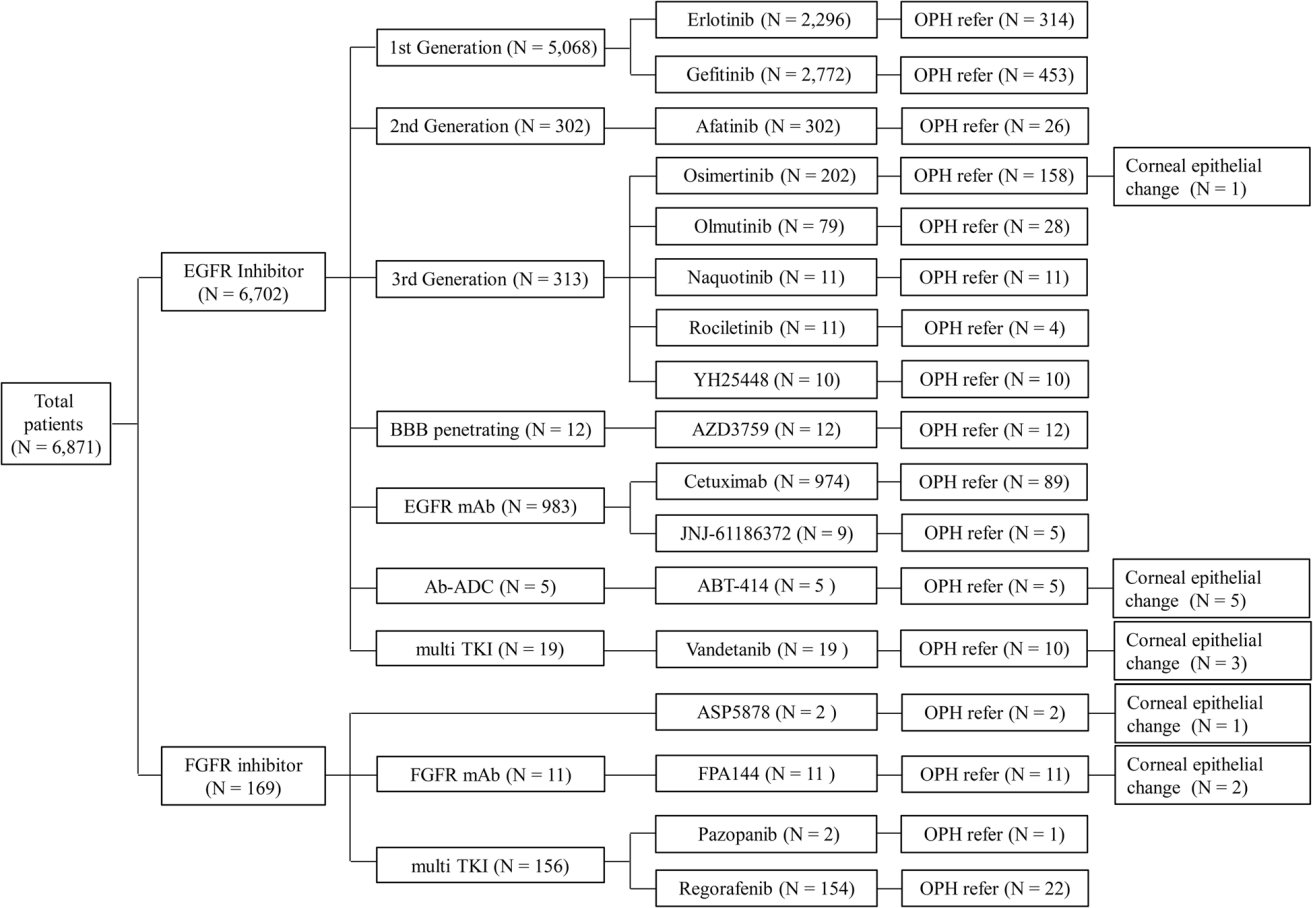
EGFR and FGFR inhibitors and their corneal epithelial changes. EGFR = epidermal growth factor receptor, FGFR = fibroblast growth factor receptor, OPH = ophthalmology department, BBB = blood-brain barrier, mAb = monoclonal antibody, Ab-ADC = antibody-drug conjugate, TKI = tyrosine kinase inhibitor

There were 6871 patients who received chemotherapy with any of following 17 agents: 13 EGFR inhibiting agents including erlotinib (Tarceva®, 150 mg oral, once daily; OSI Pharmaceuticals Inc., Melville, NY, USA and Genentech, Inc., San Francisco, CA, USA), gefitinib (Iressa®, 250 mg oral, once daily; AstraZeneca Pharmaceuticals, Cambridge, UK), afatinib (Gilotrif®, 40 mg oral, once daily; Boehringer Ingelheim, Ingelheim am Rhein, Germany), osimertinib (Tagrisso™, 80 mg oral, once daily; AstraZeneca Pharmaceuticals. Cambridge, UK), olmutinib (Olita®, 80 mg oral, once daily; Hanmi Pharm. Co., Seoul, Korea), lazertinib (YH-25448, 20 to 320 mg oral, once daily; Genosco Inc., Cambridge, MA, USA), naquotinib (ASP-8273, 300 mg oral, once daily; Astellas Pharma Inc., Tokyo, Japan), rociletinib (CO-1686, 500 mg oral, twice daily; Clovis Oncology, Inc., Boulder, CO, USA), AZD-3759 (200 mg oral, twice daily; AstraZeneca Pharmaceuticals, Cambridge, UK), cetuximab (Erbitux®, 250 mg/m^2^, intravenous infusion; Eli Lilly and Co. Indianapolis, IN, USA), JNJ-61186372 (140 mg, intravenous infusion; Genmab, København, Denmark), ABT-414, and vandetanib (Caprelsa®, 300 mg oral, once daily; AstraZeneca Pharmaceuticals, Cambridge, UK). Four FGFR-inhibiting agents included ASP-5878, FPA-144, pazopanib (Votrient™, 800 mg oral, once daily; GlaxoSmithKline, Brentford, UK), regorafenib (Stivarga®, 160 mg oral, once daily; Bayer Pharmaceuticals, Leverkusen, Germany). A total of 3669 patients (53.40%) were male while the others were female (46.60%). Additionally, 1161 patients (16.9%) referred to the ophthalmology department. Among them, 469 patients were male (40.40%) and 692 patients were female (59.60%). Finally, 12 patients had records of definite corneal epithelial changes with visual impairment after chemotherapy, while four had nonsmall-cell lung cancer (NSCLC) and were using vandetanib and osimertinib; five had glioblastoma and were using ABT-414; one had hepatocellular carcinoma (HCC) and was using ASP-5878; and two had gastric cancer and were using FPA-144. ABT-414, ASP-5878, and FPA-144 are novel drugs in a clinical trial. Figure 1 summarizes all patients prescribed any EGFR or FGFR inhibiting chemotherapeutic agents and the corneal epithelial changes.

### Ocular complications

Among 6871 patients, 1161 patients (16.90%) were referred to the ophthalmology clinic. Patients were categorized into three groups and subdivided according to their clinical diagnosis. The three groups were disease-unrelated, disease-related, and treatment-related. Patients in the disease-unrelated group had ophthalmologic diseases that were not related to their cancers and usually had minimal follow-up. The most common reasons of referral were a nonspecific regular eye check-up (Table 1) unrelated with the patient’s cancer. Among those, anterior segment problems included dry eye syndrome (6.98%), meibomian gland dysfunctions (2.67%), keratitis (1.98%), conjunctivitis (1.38%), and blepharitis (1.21%). Most of the corneal problems were without distinct corneal epithelial change and were managed with generalized treatment.

**Table 1:** Ocular complications of patients who underwent EGFR of FGFR inhibitors chemotherapy

	External adnexa					Internal Adnexa			non specific^i^	Total
	orbit^a^	eyelid^b^	sclera / conjunctiva^c^	extraocular muscles^d^	cornea^e^	lens^f^	vitreous/retina^g^	optic disc^h^		
Disease-unrelated	5	107	29	48	101	189	215	118	300	1112
Disease-related	5	–	–	–	–	–	28	–	–	33
Treatment-related	–	–	–	–	13	–	3	–	–	16
Total	10	107	29	48	114	189	246	118	300	1161

Patients were categorized into three groups and subdivided according to their clinical diagnosis. Three groups were 'Disease-unrelated', 'Disease-related' and 'Treatment-related' group

^a^Disease-unrelated (blow out fracture), Disease-related (orbit metastasis)

^b^Disease-unrelated (meibomian gland dysfunction, blepharitis, nasolacrimal duct obstruction, ptosis, dermatochalasis, cellulitis, eyelid cancer, entropion, chlazion, trichiasis)

^c^Disease-unrelated (allergic conjunctivitis, viral conjunctivitis)

^d^Disease-unrelated (strabismus)

^e^Disease-unrelated (dry eye syndrome, opacity, scar, pseudophakic bullous keraopathy, dystrophy, herpetic keratitis, exposure keratitis, epidermic keratoconjuncitivitis), Treatment-related (corneal epithelial changes, corneal ulcer)

^f^Disease-unrelated (cataract)

^g^Disease-unrelated (diabetic retinopathy, macular hole, epiretinal membrance, myopic tractional maculopathy, central serous chorioretinopathy, retinal detachmentage related macular degeneration, vitreous floater, endophthalmitis), Disease related (choroidal metastasis), Treatment-related (radiation retinopathy)

^h^Disease-unrelated (glaucoma, optic neuropathy, visual field defect)

^i^This category includes patients who had regular ophthalmologic examination without any specific lesions

Patients of other groups underwent regular follow-ups due to ophthalmologic complications. Thirty-three patients (2.84%) were in the disease-related group and had metastasis of their primary cancer to the orbit or choroid (Table 1). Sixteen patients (1.38%) in the treatment-related group had chemotherapy-related ocular complications (Table 1). One of them had a corneal ulcer, which is a rare known side-effect of erlotinib [1, 4]. Three had radiation retinopathy and 12 patients (1.03%) had definite corneal epithelial changes after treatment and had continuous follow-ups with the ophthalmology department (Table 1).

**Table 2:** EGFR and FGFR inhibitors and the incidence of corneal subepithelial changes

	Classification	Drug	Description	Total prescribed patients	patients referred to OPH (%)	Patients With corneal epithelial changes
EGFR inhibitor	1st Generation	Erlotinib	EGFR TKI which binds in a reversible fashion to the ATP binding site of the receptor	2296	314 (13.68)	0
		Gefitinib	EGFR TKI which binds to ATP binding site of the receptor	2772	453 (16.34)	0
	2nd Generation	Afatinib	selectively and irreversibly binds to and inhibits the EGFR 1, 2, and 4, and certain EGFR mutants, including those caused by EGFR exon 19 deletion mutations or exon 21 (L858R) mutations.	302	26 (8.61)	0
	3rd Generation	Osimertinib	a third generation EGFR inhibitor which shows 200-fold selectivity for T790 M/L858R protein over wild-type EGFR	202	158 (78.22)	1 (0.50%)
		Olmutinib	EGFR mutation-specific tyrosine kinase inhibitor	79	28 (35.44)	0
		YH25448	a Highly Selective third Generation EGFR TKI	10	10 (100.00)	0
		Naquotinib	binds to and inhibits the activity of mutant forms of EGFR, including the T790 M EGFR mutant	11	11 (100.00)	0
		Rociletinib	inhibits T790 M, a secondary acquired resistance mutation, as well as other mutant EGFRs	11	4 (36.36)	0
	BBB penetrating	AZD3759	a novel EGFR tyrosine kinase inhibitor with high capability to penetrate the BBB	12	12 (100.00)	0
	EGFR mAb	Cetuximab	a chimeric monoclonal antibody which binds to and inhibits EGFR	974	89 (9.14)	0
		JNJ-61186372	bispecific antibody that targets EGFR and cMet	9	5 (55.56)	0
	Ab-ADC	ABT-414	an investigational compound targeting a tumor-selective EGFR epitope	5	5 (100.00)	5 (100.0%)
	multi TKI	Vandetanib	a selective inhibitor of EGFR and vascular endothelial growth factor receptor 2 (VEGFR2) tyrosine kinase	19	10 (52.63)	3 (15.79%)
FGFR inhibitor		ASP5878	a novel drug which inhibits all FGFRs targeting FGF19-expressing HCC	2	2 (100.00)	1 (50.0%)
	FGFR mAb	FPA144	an enhanced Monoclonal Antibody against FGFR2b	11	11 (100.00)	2 (18.18%)
	multi TKI	Pazopanib	a potent and selective multi-targeted receptor tyrosine kinase inhibitor that blocks tumour growth and inhibits angiogenesis (inhibits VEGFR-1, VEGFR-2, VEGFR-3, PDGFR-alpha, beta receptors, cytokine receptor)	2	1 (50.00)	0
		Regorafenib	Multikinase inhibitor (VEGFR 1–3, PDGFR-alpha, PDGFR-beta, etc)	154	22 (14.29)	0

*EGFR* epidermal growth factor receptor, *ATP* adenosine triphosphate, *FGFR* fibroblast growth factor receptor, *OPH* ophthalmology department, *BBB* blood-brain barrier, *mAb* monoclonal antibody, *cMet* tyrosine-protein kinase Met, *Ab-ADC* antibody-drug conjugate, *TKI* tyrosine kinase inhibitor, *VEGFR* Vascular Endothelial Growth Factors, *PDGFR* Platelet-derived growth factor receptor

### Corneal epithelial changes with visual distortion

Table 2 present the demographics and chart review results of the 12 patients with corneal changes. Five chemotherapy agents (vandetanib, osimertinib, ABT-414, ASP-5878, and FPA-144) were related to corneal epithelial lesions.

**Fig. 2:**
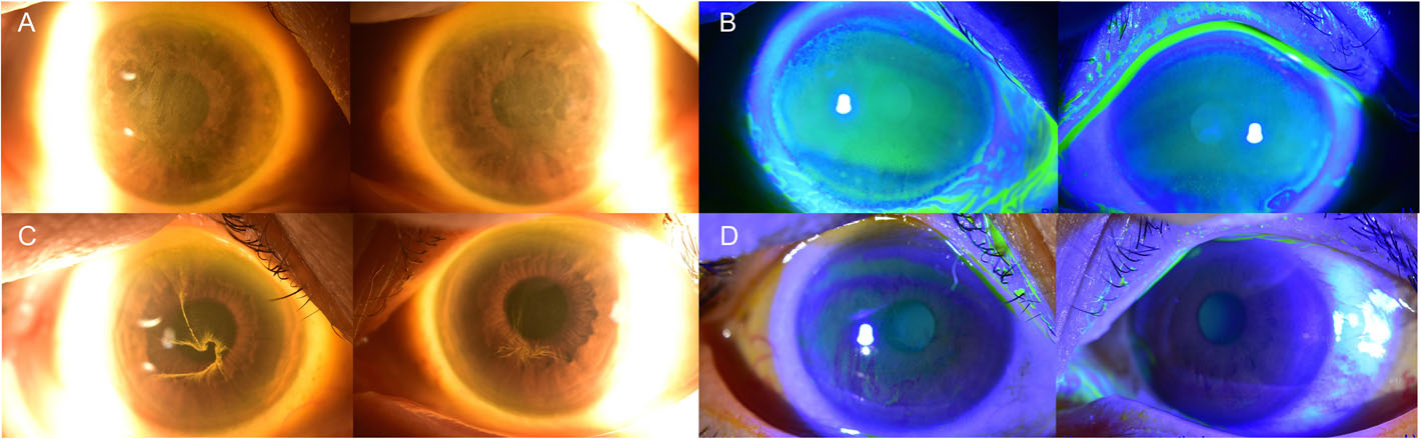
Anterior segment photographs of patients on vandetanib and osimertinib. Corneal photographs of case 2 taken at 419 days after the start of chemotherapy with vandetanib. **a** Both corneas showed dense cornea verticillata on the central part (yellow arrows Δ). **b** Under fluorescein staining, no corneal epithelial defects were found. Corneal photographs of case 4 taken at 305 days after start of chemotherapy with osimertinib. **c** Vortex keratopathy with a whorl-like pattern was prominent, especially on the patient’s right cornea (yellow arrows Δ). **d** Under fluorescein staining, no corneal epithelial defects were found

Among 19 patients with vandetanib, a selective inhibitor of EGFR and vascular EGRF 2 tyrosine kinase [10], three patients showed vortex keratopathy (Fig. 2a and b). One among 202 patients with osimertinib, a third-generation EGFR inhibitor which shows 200-fold selectivity for the T790 M/L858R protein over wild-type EGFR [11], also had vortex keratopathy (Fig. 2c and d). The other five were patients with glioblastoma who received chemotherapy with ABT-414. The incidence of corneal epithelial changes among all patients treated was 15.79% with vandetanib, 0.5% with osimertinib, and 100% with ABT-414 (Table 2). Both vandetanib and osimertinib were recently approved by the Food and Drug Administration (FDA) of United States, while ABT-414 is an investigational drug undergoing clinical trials.

**Fig. 3:**
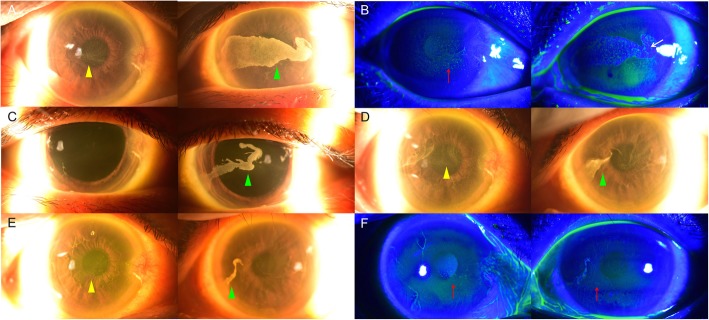
Anterior segment photographs of a patient (case 7) on ABT-414. **a** Images taken 317 days after the first injection (total of 21 shots), where the patient’s right cornea shows a whorl-like vortex keratopathy (yellow arrow Δ) and the left cornea shows dense central keratopathy (green arrow Δ). **b** Under fluorescein staining, punctate epithelial erosions (red arrow↑) were found along the vortex keratopathy of the right eye. Fluorescein dye was blocked by the dense central keratopathy (white arrow↑), while a few punctate epithelial erosions were found along the central lesion in the left eye. **c** At 65 days after drug discontinuation, the right cornea seemed almost clear and the left corneal lesion was much thinner (green arrow Δ). **d** A newly developed central whorl-like vortex keratopathy was observed after the patient received eight more injections 2 months after the images in (**c**) were taken (yellow arrow Δ and green arrow Δ). **e** Both corneas showed much thinner keratopathy 2 months after the second instance of discontinuation of the drug (yellow arrow Δ and green arrow Δ). **f** Although a few punctate epithelial erosions (red arrow↑) were seen along the vortex keratopathy, it was much clearer as compared with at the time of recording (**b**)

The mean duration of chemotherapy was 309 days for patients on vandetanib and 152 days with ABT-414. The mean total dose of vandetanib was 3500 mg in three patients, while, for ABT-414, it was 832.33 mg in five patients. The patient with osimertinib had continuously taken 80 mg (1 tablet) of the drug orally since January 13, 2017. The mean interval between the initiation of chemotherapy and the diagnosis of a corneal epithelial lesion was 246 days with vandetanib, but only 30 days with ABT-414, which was much shorter than that for the other drugs. Specific intervals and durations of the drugs in each patient are described in Table 3. The mean accumulated drug dose at the time of corneal lesion diagnosis was 2800 mg for vandetanib, 15,680 mg for osimertinib, and 221.77 mg for ABT-414. Vortex keratopathy, with a whorl-like pattern of corneal haziness, was found in nine of the patients described above. During the early stages, the lesion started from the superior or inferior border of the cornea and spread to the center. As the duration of chemotherapy lengthened, cornea verticillata became much clearer (Figs. 2 and 3).

**Table 3:** Demographics and chart review of patients who developed corneal epithelial changes

	Case	Drug	Sex	Age	Diagnosis	Corneal Features	Duration of chemotherapy (day)	Interval 1 (day)	Interval 2 (day)	Visual acuity before chemotherapy (log MAR)		Lowest visual acuity after chemothearpy (log MAR)		Highest visual acuity after end of therapy (log MAR)		Corneal clearance
										OD	OS	OD	OS	OD	OS	
EGFR inhibitor (Tyrosine Kinase Inhibitor)	1	Vandetanib	2	30–39	NSCLC	Vortex Keratopathy	284	91	Deceased	0	0	0	0	0	0	N/A
	2		2	60–69	NSCLC		390	395	F/u loss	0.097	0.097	0.301	0.155	0.097	0.097	partial
	3		2	70–79	NSCLC		253	252	230	0.155	0.155	0.222	0.301	0.222	0.155	total
	4	Osimertinib	2	50–59	NSCLC		Ongoing	196	Ongoing	0	0	0.155	0.155	N/A	N/A	N/A
	5	ABT-414	1	50–59	Glioblastoma		59	35	59	0	1	1.222	2	0.699	0.523	partial
	6		2	70–79	Glioblastoma		231	49	141	0.046	0.301	0.523	0.523	0	0.097	total
	7		2	50–59	Glioblastoma		347	23	181	0	0	0.523	0.301	0	0	total
	8		1	40–49	Glioblastoma		111	22	79	0	0	0.301	0.222	0	0	total
	9		2	60–69	Glioblastoma		14	22	149	0	0	0.301	0.699	0	0	total
FGFR inhibitor	10	ASP5878	2	40–49	HCC	Corneal epithelial changes mimicking dysmaturation	195	55	93	0	0	0.301	0.155	0	0	total
	11	FPA144	2	40–49	Gastric cancer		459	73	218	0	0	0.046	0.398	0	0	total
	12		2	60–69	Gastric cancer		70	68	F/u loss	0	0	0.155	0.046	0.097	0.046	partial

*EGFR* epidermal growth factor receptor, *FGFR* fibroblast growth factor receptor, *NSCLC* Non-small cell lung cancer, *HCC* Hepatocellular carcinoma, *Interval 1* Interval between start of chemotherapy and diagnosis of corneal change (day), *Interval 2* Interval between end of chemotherapy and corneal recovery (day), *OD* Oculus Dextra (right eye), *OS* Oculus Sinistra (left eye), *logMAR* logarithm of minimal angle of resolution units, *N/A* Not applicable

All patients on EGFR inhibitors complained of progressive visual acuity deterioration after the development of corneal lesions. Full recovery from corneal epithelial changes were confirmed in one patient on vandetanib 230 days following the discontinuation of the drug and in four patients with ABT-414 at an average of 122 days after the end of chemotherapy (Table 3). One patient with vandetanib and one patient with ABT-414 showed partial recovery of the corneal epithelium (Table 3). At the end of the trial, one of the patients taking vandetanib was deceased and another was lost to follow-up. Additionally, a patient on osimertinib is still undergoing chemotherapy, so we could not clarify the clearance of vortex keratopathy after discontinuation of the drug. In patients with full recovery of corneal epithelial lesions, highest visual acuity in logarithm of minimal angle of resolution units (logMAR) after discontinuation of chemotherapy were 0.044 ± 0.089 in right eye and 0.050 ± 0.064 in left eye. Highest logMAR visual acuity of patients with partial recovery of cornea after discontinuation of agents were 0.398 ± 0.301 in right eye and 0.310 ± 0.213 in left eye.

Three patients showed corneal epithelial changes after using FGFR inhibitors. The incidence rates of corneal epithelial changes among all treated patients were 50.0% with ASP-5878 and 18.18% with FPA-144. Both were, as described, novel drugs. The patient with HCC received chemotherapy with ASP-5878 for 195 days. Two patients with gastric cancer received chemotherapy with FPA-144 for 264 days on average. The total dose was 4704 mg for ASP-5878 and the mean total dose was 8660 mg for FPA-144. The interval between the initiation of chemotherapy and diagnosis of corneal lesions was 55 days with ASP-5878 and 70 days on average with FPA-144. Specific intervals and durations of the drugs in each patient are described in Table 3. The accumulated dose at the diagnosis of corneal lesion was 1320 mg in ASP-5878 and mean accumulated dose of FPA-144 at the diagnosis of corneal lesion was 3163 mg.

**Fig. 4:**
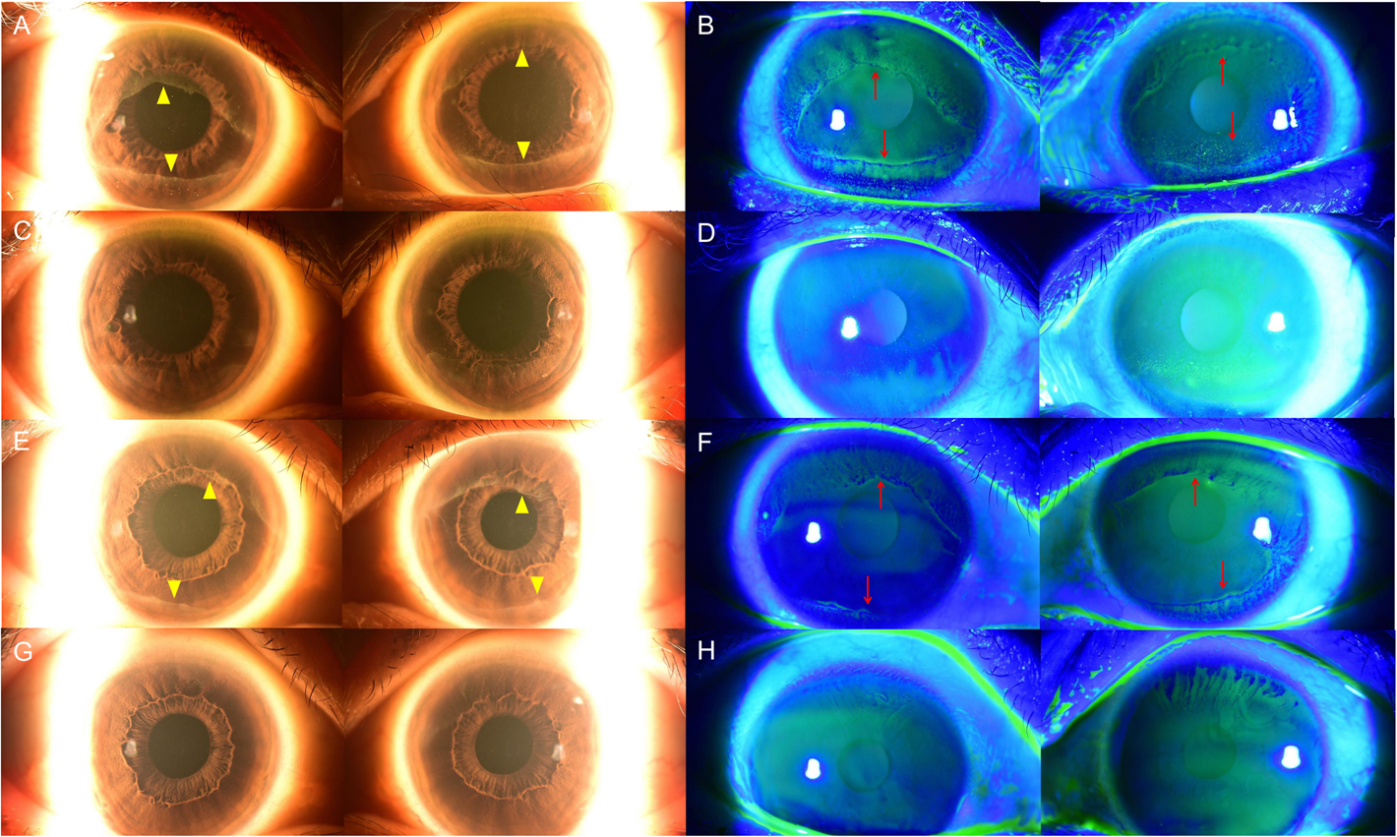
Anterior segment photographs of two patients on FGFR inhibitors. Anterior segment photograph of case 10 patient. **a** The cornea of case 10 showed diffuse opacification, leaving the central part intact (yellow arrows Δ) at 190 days after the start of ASP-5878. **b** Unlike patients with EGFR inhibitor chemotherapy, this patient’s cornea showed epithelial staining along the demarcation of keratopathy (red arrows↑). **c** At 93 days after the discontinuation of the drug, both corneas were much improved and only thin opacification remained. **d** Under fluorescein staining, the epithelial staining appeared almost gone, leaving small peripheral lesions. Anterior segment photograph of case 11 patient. **e** At 446 days after the first intravenous injection of FPA-144, both corneas showed similar keratopathy findings, with superior and inferior demarcations (yellow arrows Δ). **f** Under fluorescein staining, there was corneal epithelial staining noted beyond the demarcation line (red arrows↑). **g** AT 218 days after the discontinuation of the drug, both corneas were considerably cleared. **h** Under fluorescein staining, the peripheral part of the left cornea showed epithelial staining that was much improved when compared with in (**f**)

Corneal changes in the three patients after FGFR inhibitor chemotherapy resembled clinical features of corneal dysmaturation, showing an opalescent epithelium without fibrovascular corneal pannus (Fig. 4). All patients with FGFR inhibitors complained of decreased visual acuity after the development of a corneal lesion. Recovery of visual acuity and corneal changes were confirmed for two patients. One patient of FPA-144 was lost to follow-up. More detailed descriptions of the corneal features are presented in Fig. 4. In two patients with full recovery of corneal epithelial lesions, highest log MAR visual acuity after discontinuation of chemotherapy were 0.000 ± 0.000 in both eyes. Highest logMAR visual acuity of the patient with partial recovery of cornea after discontinuation of agents were 0.097 in right eye and 0.046 in left eye.

## Discussion

We found that three among the 19 patients (15.79%) on vandetanib, one among the 202 patients (0.50%) on osimertinib, and all five patients (100%) on ABT-414 showed vortex keratopathy. All patients except those deceased shortly after the initiation of chemotherapy complained of decreased visual acuity due to corneal epithelial changes. While old drugs showed a low incidence of corneal change causing vision deterioration, more recently used novel drugs in clinical trials revealed obviously high incidence rates of ocular side effects. Fortunately, full recovery of both vision and the cornea was confirmed in seven patients (Table 3).

Vortex keratopathy is a condition characterized by a whorl-like pattern of corneal deposits in the corneal epithelium. Well-known causes of the disease are amiodarone use or Fabry’s disease, which is also known as lysosomal storage disorder [5]. Beyond amiodarone, other drugs that are known to cause vortex keratopathy include vandetanib and osimertinib [5]. There have been few case reports of vortex keratopathy after EGFR inhibitor chemotherapy reported to date. Ahn et al. reported a case of vortex keratopathy after six one-month cycles of 300 mg/day of vandetanib [10]. Chia et al. reported vortex keratopathy presumed to develop 8 months after the use of AZD9291, a third-generation tyrosine kinase inhibitor (TKI) currently known as osimertinib. Both case reports did not address the prognosis of the corneal lesions [12]. We reviewed a tertiary hospital database for patients who used any kind of EGFR or FGFR inhibitors. Although there could be some variation depending on the geographic region, country, and institution, we calculated the incidence of vortex keratopathy for each of the drugs. Furthermore, we confirmed that the keratopathy cleared or at least improved after discontinuation of the offending agents.

Drug-induced vortex keratopathy is typically associated with cationic and amphiphilic medications that penetrate lysosomes and bind to cellular lipids. It is believed that drug–lipid complexes are the intra-lysosomal inclusion bodies observed in the basal layers of the corneal epithelium [10, 13]. The mechanism of vortex keratopathy with EGFR receptor inhibitors was thought to be either deposits of chemotherapy agent-derived metabolites in the cornea or abnormal turnover or migration of corneal epithelial cells due to the inhibition of corneal EGFR [5]. Recently, interactions between vandetanib and lysozymes and their characteristics were described, providing support for the previously suggested role of drug metabolites [14].

Strikingly, every patient who used ABT-414 showed vortex keratopathy. This is quite alarming given that there were three cases out of 19 patients on vandetanib and one out of 192 patients on osimertinib in comparison who showed the same. Moreover, in two cases, vortex keratopathy developed within only 22 days after the first infusion of the drug. In the AbbVie study, patients received prophylactic steroid eye drops three times a day for a week starting 2 days prior to each infusion and continuing for 4 days after. According to the phase I study of ABT-414, patients showed dose-related ophthalmologic toxicity outcomes such as dry eyes, blurry vision, eye pain, photophobia, watery eyes, and findings of microcyst development within the cornea. Although it is unclear whether this microcystic development refers to vortex keratopathy, the article suggested that steroid eye drops could be used to help reduce the incidence and severity of those side effects [6]. Despite previous reports on the usefulness of prophylactic steroid eye drops in reducing the incidence of ocular side effects from ABT-414 [6, 15], this treatment did not seem effective in preventing the development of vortex keratopathy in our study, since all five patients with ABT-414 had developed such corneal lesions.

ABT-414 is an antibody-drug conjugate (ADC) consisting of three components: an EGFR-targeting humanized monoclonal antibody, a potent microtubule agent (monomethyl auristatin F [MMAF]) and a noncleavable maleimidocaproly linker that connects MMAF to the antibody [7]. In clinical development, ABT-414 was designed to limit binding to wild-type EGFR [15]. Therefore, MMAF is supposed to have a lower cytotoxicity, attenuated potency, and improved aqueous solubility as compared with its uncharged counterpart, monomethyl auristatin E (MMAE) [16].

However, the reduced toxicity does not appear to extend to ocular toxicity. According to a review article on ADCs with MMAF and MMAE published in 2015, there were five cases of corneal microcystic epithelial changes and four cases of corneal deposits or inclusions [17]. Interestingly, among the list of ADCs associated with ocular side effects, four of 13 used MMAF as a cytotoxin. However, none of the ADCs employing MMAE as a cytotoxin were associated with ocular side effects [17].

MMAF, an antimitotic auristatin derivative with a charged C-terminal phenylalanine residue [16], is a microtubule inhibitor that induces apoptosis in cells undergoing mitosis. However, a recent study showed that it may also disrupt nondividing cells in interphase [15]. Although it is not clear as to why MMAF preferentially disrupts corneal cells, the ocular toxicity of the substance seems to have promoted more instantaeneous formation of vortex keratopathy following dosing with ABT-414.

Despite the extensive number of medical records of this study, conventional EGFR inhibitors such as erlotinib, geftinib, afatinib, and cetuximab were not linked with corneal epithelial changes in any cases. There were few patients who had chart records of simple punctate epithelial erosions and none of these had evidence of vortex keratopathy. Although the mechanisms are not clear, the latest EGFR inhibitors seem to affect the corneal epithelium more directly than conventional drugs.

Although we could not calculate the accurate incidence of vortex keratopathy following the use of EGFR inhibitors as chemotherapy due to the nature of this study, the condition does not seem uncommon when dealing with recently developed agents. Vandetanib is a second-generation EGFR inhibitor and osimertinib is a third-generation EGFR inhibitor that can target T790 M and EGFR TKI-sensitizing mutations while sparing wild-type EGFR [18]. ABT-414 is an investigational compound. Quite a few patients developed vortex keratopathy after using these three chemotherapy agents. There were also some new or investigational drugs that did not promote vortex keratopathy in patients such as olmutinib, naquotinib, rociletinib, AZD-3759, JNJ-61186372 and regorafenib. However, there is a possibility that lesions in conjunction with the use of these drugs could be found in a much larger cohort.

On the other hand, we suspected corneal dysmaturation in patients who used FGFR inhibitors. Corneal dysmaturation is a benign and indolent condition that leads to a frosted corneal epithelium or individual islands of opalescent epithelium. Fibrovascular corneal pannus is not present [19]. Unlike vortex keratopathy, corneal dysmaturation is scarcely reported in the literature, which complicated our ability to obtain evidence. Furthermore, although histological findings are important for accurate diagnosis, we did not have access to samples of corneal lesions since this was a retrospective study. Nevertheless, the opacification pattern of patient with FGFR inhibitor agent was different from that of vortex keratopathy and was clearly distinguishable upon comparison with that of EGFR inhibitors. As there is no supporting evidence of histopathologic specimens among our patients, we cannot rule those with keratopathy after FGFR inhibitor chemotherapy as having corneal dysmaturation. However, there have not been any reports regarding corneal changes associated with the use of FGFR inhibitors to the best of our knowledge. Therefore, our findings are meaningful that we found out the clinical features were different from vortex keratopathy. It would be unwise to draw conclusions from our study that actual incidence rates of this corneal epithelial change after FGFR inhibitor treatment are as high as we suggested since the total number of patients included was too small.

We also reviewed the records of NSCLC patients on regorafenib, a multikinase inhibitor that blocks FGFR1 and 2 and various other receptors, and pazopanib, a multikinase inhibitor that blocks FGFR, vascular endothelial growth factor receptor, and platelet-derived growth factor receptor, but did not find any cases similar to those of the three patients on FGFR inhibitors. We hypothesize that selective strong affinities of ASP-5878 and FPA-144 toward FGFR affected corneal changes in the three patients. Since we could not find any report describing ophthalmological findings following FGFR inhibitor chemotherapy, further research on the influences of FGFR inhibitors on the corneal epithelium is needed.

It is difficult to define the exact time lapse between the start of chemotherapy and the appearance of corneal epithelial changes because not all patients underwent regular follow-up examinations during and after chemotherapy. However, it is clear that these corneal lesions are able to develop quite fast, suggesting the possibility that previously reported cases might have been diagnosed far later than at the time of the actual onset of keratopathy. One of our cases on vandetanib developed vortex keratopathy 91 days after the first chemotherapy session, which is much quicker than previously reported. Separately, one case on ASP-5878 took only 55 days to develop a corneal lesion, while two patients on FPA-144, an enhanced monoclonal antibody against FGFR2b, took 2 months. Furthermore, three patients in the ABT-414 group took less than 1 month to develop vortex keratopathy.

All patients who developed corneal epithelial changes were treated with recommended regimens as follows. According to the FDA of United States, the recommended dose of osimertinib is 80 mg in tablet form once a day until disease progression or unacceptable toxicity. In ABT-414, the recommended regimen is 1.25 mg/kg via intravenous infusion every 2 weeks over 30 to 40 min [6, 20]. In clinical studies of vandetanib, patients received vandetanib 300 mg once daily [21, 22]. In clinical trials of ASP-5878, varying oral doses of 2 mg twice daily to 20 mg twice daily were given to patients [8]. In our center, 12 mg twice daily was given orally. The recommended dose of FPA-144 is 15 mg/kg given by intravenous infusion every 2 weeks [23]. Although it is not clear, it seems that there are certain accumulated doses of drugs that evoke corneal epithelial changes as patients complained of decreased visual acuity after the lapse of certain length of time. As seven patients achieved full recovery and three showed partial recovery from corneal epithelial changes after discontinuation of the drugs, the decrease of accumulated dose of chemotherapy agents might be important for the prognosis of corneal lesions.

It is important to check which other chemotherapy agents the affected patients in the study took before receiving EGFR or FGFR inhibitors elucidate whether these agents had any influence in the development of corneal epithelial lesions. Table 4 presents prior chemotherapy agents used before EGFR and FGFR inhibitor therapy in the affected patients. First, considering geftinib and afatinib, we did not find evidence of vortex keratopathy in this study as we discussed and there was no report of such among the literature. Second, we reviewed the literature dealing with ocular side effects of the listed chemotherapy agents in Table 4. Although quite a few agents listed above had a variety of ocular side effects, none of them had any evidence specifically dealing with corneal epithelial changes (Table 5). Moreover, vandetanib and osimertinib already have collected a few reports between them on the topic of corneal epithelial changes. Therefore, we concluded it is reasonable to believe that the observed corneal epithelial changes that occurred in 12 patients in this study were induced by the EGFR or FGFR inhibitors highlighted.

**Table 4:** Prior chemotherapy agents before EGFR and FGFR inhibitor therapy in affected patients

	Case	Drug	Sex	Age	Diagnosis	Prior chemotherapy agents before study drugs
EGFR inhibitor (Tyrosine Kinase Inhibitor)	1	Vandetanib	2	30–39	NSCLC	Doxorubicin, Cisplatin, Pemetrexed, Geftinib, Gemcitabine
	2		2	60–69	NSCLC	Doxorubicin, cisplatin
	3		2	70–79	NSCLC	Gemcitabine, cisplatin
	4	Osimertinib	2	50–59	NSCLC	Afatinib
	5	ABT-414	1	50–59	Glioblastoma	Temozolomide
	6		2	70–79	Glioblastoma	Temozolomide
	7		2	50–59	Glioblastoma	Temozolomide
	8		1	40–49	Glioblastoma	Temozolomide
	9		2	60–69	Glioblastoma	none
FGFR inhibitor	10	ASP5878	2	40–49	HCC	Sorafenib, Ramucirumab, Everolimus
	11	FPA144	2	40–49	Gastric cancer	Capecitabine, Oxaliplatin, Paclitaxel, Doxorubicin, Cisplatin, Irinotecan
	12		2	60–69	Gastric cancer	Gemcitabine, Cisplatin, Doxorubicin, Capecitabine, Oxaliplatin

*EGFR* epidermal growth factor receptor, *FGFR* fibroblast growth factor receptor, *NSCLC* Non-small cell lung cancer, *HCC* Hepatocellular carcinoma

**Table 5:** Known ocular side effects of prior chemotherapy agents before EGFR and FGFR inhibitor therapy

Prior chemotherpy agents before study drugs	Mechanism [24–32]	Known ocular side effects
Afatinib	EGFR-inhibitor	Anterior uveitis [32]
Capecitabine	Antimetabolite/pyrimidine analog	dry eye, lacrimation [24],
Cisplatin	Alkylating agent/platinum	Blurred vision, retinotoxicity (retinal ischaemia and neovascularization), optic neuropathy [33]
Doxorubicin	anthracyline drug	conjunctivitis (m/c), periorbital edema, lacrimation, blepharospasm, keratitis, and decreased visual acuity [34]
Everolimus	mTOR inhibitor	posterior reversible encephalopathy syndrome,bilateral optic neuropathy [35]
Geftinib	EGFR-inhibitor	Dry eye, corneal ulcer [36, 37]
Gemcitabine	antimetabolite agent	uveal effusion and outer retinal disruption or Purtscher-like retinopathy with combination of docetaxel [25, 38]
Irinotecan	topoisomerase I inhibitor	None [39], none in combination regimen with 5-fluorouracil, oxaliplatin and bevacizumab [40]
Oxaliplatin	platinum	dry eye, lacrimation, conjunctivitis, damage of the retinal pigment epithelium and the optic nerve, visual field defect [30, 41]
Paclitaxel	Antimicrotubule agent	epiphora, eye pain, conjunctivitis, blepharitis, blurred vision [24, 42]
Pemetrexed	Antimetabolite /antifolate	AION [1], eyelid edema [24, 43]
Ramucirumab	monoclonal Ab of VEGFR-2	none [28]
Sorafenib	anti-VEGF agent	blurred vision, periocular edema, retinal detachment, ptosis, optic neuropathy, macular edema [27, 44]
Temozolomide	alkylating/methylating agent	blurred vision with concurrent radiation therapy [45].

*AION* Anterior ischemic optic neuropathy, *VEGF* Vascular endothelial growth factor, *Ab* Antibody, *VEGFR* Vascular endothelial growth factor receptor, *mTOR* mammalian target of rapamycin

Two cases on vandetanib and one case on osimertinib experienced a delay in reaching a diagnosis of ocular complications. However, this delay may have been due to late consultation with the ophthalmology department, as these patients did not have regular ophthalmologic examinations before they complained of decreased visual acuity. In our study, among 6871 patients, only 16.89% had any record of ophthalmologic examinations. Furthermore, only one-third of these participated in a consultation or follow-up visit associated with their chemotherapy treatment. The rest of the medical records did not relate to the patients’ chemotherapy.

Lack of knowledge of corneal epithelial changes after the use of EGFR or FGFR inhibitors among clinicians may cause them to disregard the importance of consultation with the ophthalmology department before, during, and after chemotherapy. Moreover, visual impairment may not be considered important when compared with other systemic side effects of chemotherapy. Therefore, we are unable to estimate how many patients may have experienced symptoms without referral or diagnosis.

Furthermore, due to the nature of the study, patients were detected by retrospective chart review, which allows for possible selection bias. Subtle symptoms and clinical findings may have been caused by these agents yet may have been described as “puntate epithelial erosions or dry eyes,” which were not included as cases. Therefore, the percentages of patients affected by a given agent do not accurately summarize the true prevalence of ocular toxicity associated with that agent. In other words, the actual incidence of corneal epithelial changes after EGFR or FGFR chemotherapy could have been underestimated.

Another limitation of our study is a possibility that actual causes of corneal epithelial changes could be more complicated. The drugs discussed here are varied, with different mechanisms of action. Some results could be off-target effects or could be affected by other drugs that the patient took during chemotherapy. However, vortex keratopathy associated with osimertinib, vandetanib, and ABT-414 was already reported in previous articles. Also, ASP-5878 and FPA-144 are not multi-TKI. Therefore, the results of our study, although somewhat inaccurate, have significance in that we examined long-term follow-up records and confirmed a full recovery of keratopathy in many cases.

In our study, steroid eye drops were not effective in preventing corneal epithelial changes after treatment with ABT-414. Nevertheless, all patients showed improved visual acuity and corneal surfaces after discontinuing the drug. Reardon et al. also pointed out that, once ABT-414 treatment was held or discontinued, ocular symptoms gradually resolved spontaneously in the majority of patients. They speculated that the corneal epithelium would regenerate after discontinuation of ABT-414, eliminating microcysts caused by the drug [6]. Therefore, considering the possible side effects of steroid eye drops, it is wise not to use them for prophylaxis. Overall, patients undergoing chemotherapy with EGFR or FGFR inhibitors should be educated about the possibility of corneal epithelial changes that reduce visual acuity and reassured that the condition is generally reversible after the end of treatment.

## Conclusions

Chemotherapy using EGFR or FGFR inhibitors can cause corneal epithelial changes with decreased visual acuity that recovers following discontinuation of the agents. Unfortunately, some physicians remain unaware of such side effects, leaving many patients unmanaged. Therefore, ophthalmologists should forewarn patients who are planning chemotherapy with such agents about the possibility of corneal changes that lead to the clouding of vision. Doctors should also clarify that the corneal lesions will probably resolve after the end of chemotherapy.

## Data Availability

The data that support the findings of this study are from medical records of Samsung Medical Center patients and were used under license for the current study, so are not publicly available.

## Abbreviations

ADC: Antibody-drug conjugate
EGF: Epidermal growth factor
EGFR: Epidermal growth factor receptor
FDA: Food and Drug Administration
FGF: Fibroblast growth factor
FGFR: Fibroblast growth factor receptor
HCC: Hepatocellular carcinoma
MMAE: Monomethyl auristatin E
MMAF: Monomethyl auristatin F
NSCLC: Nonsmall-cell lung cancer
TKI: Tyrosine kinase inhibitor
